# Hard wiring of normal tissue-specific chromosome-wide gene expression levels is an additional factor driving cancer type-specific aneuploidies

**DOI:** 10.1186/s13073-021-00905-y

**Published:** 2021-05-25

**Authors:** Sushant Patkar, Kerstin Heselmeyer-Haddad, Noam Auslander, Daniela Hirsch, Jordi Camps, Daniel Bronder, Markus Brown, Wei-Dong Chen, Rachel Lokanga, Darawalee Wangsa, Danny Wangsa, Yue Hu, Annette Lischka, Rüdiger Braun, Georg Emons, B. Michael Ghadimi, Jochen Gaedcke, Marian Grade, Cristina Montagna, Yuri Lazebnik, Michael J. Difilippantonio, Jens K. Habermann, Gert Auer, Eytan Ruppin, Thomas Ried

**Affiliations:** 1 Cancer Data Science Laboratory, Center for Cancer Research, National Cancer Institute, NIH, Bethesda, MD 20892 USA; 2 Department of Computer Science, University of Maryland, College Park, USA; 3 Section of Cancer Genomics, Center for Cancer Research, National Cancer Institute, NIH, Bethesda, MD 20892 USA; 4 National Center for Biotechnology Information, NIH, Bethesda, MD 20892 USA; 5 Gastrointestinal and Pancreatic Oncology Team, Institut D’Investigacions Biomèdiques August Pi i Sunyer, (IDIBAPS), Hospital Clínic of Barcelona, CIBEREHD, 08036 Barcelona, Spain; 6 Section for Translational Surgical Oncology and Biobanking, Department of Surgery, University Medical Center Schleswig Holstein, Campus Lübeck, Lübeck, Germany; 7 Department of General, Visceral and Pediatric Surgery, University Medical Center, Göttingen, Germany; 8 Department of Genetics and Pathology, Albert Einstein College of Medicine, Bronx, NY USA; 9 Lerna Consulting, LLC, New Haven, CT 06511 USA; 10 Division of Cancer Treatment and Diagnosis, National Cancer Institute, NIH, Bethesda, MD 20892 USA; 11 Department of Oncology and Pathology, CancerCenter Karolinska, Karolinska Institute and University Hospital, Stockholm, Sweden

## Abstract

**Background:**

Many carcinomas have recurrent chromosomal aneuploidies specific to the tissue of tumor origin. The reason for this specificity is not completely understood.

**Methods:**

In this study, we looked at the frequency of chromosomal arm gains and losses in different cancer types from the The Cancer Genome Atlas (TCGA) and compared them to the mean gene expression of each chromosome arm in corresponding normal tissues of origin from the Genotype-Tissue Expression (GTEx) database, in addition to the distribution of tissue-specific oncogenes and tumor suppressors on different chromosome arms.

**Results:**

This analysis revealed a complex picture of factors driving tumor karyotype evolution in which some recurrent chromosomal copy number reflect the chromosome arm-wide gene expression levels of the their normal tissue of tumor origin.

**Conclusions:**

We conclude that the cancer type-specific distribution of chromosomal arm gains and losses is potentially “hardwiring” gene expression levels characteristic of the normal tissue of tumor origin, in addition to broadly modulating the expression of tissue-specific tumor driver genes.

**Supplementary Information:**

The online version contains supplementary material available at 10.1186/s13073-021-00905-y.

## Background

In solid tumors of epithelial origin, i.e., carcinomas, and in certain other solid tumors such as glioblastoma multiforme and malignant melanoma, aneuploidies of specific chromosomes define the landscape of somatically acquired genetic changes [1–5]. In fact, aneuploidy is present in about 90% of solid tumors [6]. Remarkably, the distribution of ensuing genomic imbalances is cancer type-specific [4, 7]. For instance, colorectal carcinomas recurrently gain chromosome arms 7, 8q, 13q, and 20q and lose copies of 8p, 17p, and 18q [8]. In contrast, cervical carcinomas recurrently gain chromosome arms 1q and 3q. In other words, a gain of 3q is not observed in colorectal cancer, and cervical carcinomas do not have copy number gains of, e.g., chromosomes 7 or 13q [4, 5, 7]. Furthermore, cancer type-specific chromosomal aneuploidies emerge in dysplastic, i.e., not yet malignant, lesions, that are prone to progress to invasive disease [8–12]. Numerous cancer type-specific aneuploidies originate at early stages of tumorigenesis, yet are retained in late stage tumors and in metastases, as reflected in the TCGA database [9].

The cancer type-specific distribution of genomic imbalances was recently confirmed in two comprehensive pan-cancer analyses of several thousand tumors [10, 11]. Although some intra-tissue differences were observed for certain tumor subtypes arising from the same tissue, different tumor types from the same tissue tended to cluster together (e.g., low-grade gliomas cluster with glioblastomas as do clear cell and papillary renal cell carcinomas). On one hand, it is possible that loss or gain of particular chromosomes or their fragments during carcinogenesis target the gain of specific oncogenes or the loss of tumor suppressors located on these chromosomes [6, 12, 13]. On the other hand, it is well known that chromosome-wide alterations of gene expression levels follow genomic copy number changes [14, 15], i.e., the transcripts of genes that are located on gained chromosomes are more, and those on lost chromosomes are less abundant. This correlation has been firmly established in primary human carcinomas, in derived cell lines, and in experimental cancer models [14, 16–20]. Hence, the gain or loss of specific chromosomes can potentially act as a mechanism to maintain tissue-specific gene dosage.

Given this background, we decided to explore how the frequencies of chromosomal arm gains and losses in specific cancer types correlate with (i) mean chromosome arm gene expression levels of their normal tissue of origin and (ii) the chromosomal distribution of previously identified or newly implicated tissue-specific driver genes. Our exploratory analysis unearthed a complex picture of factors shaping the evolution of tumor karyotypes in which frequent chromosomal copy number changes can potentially “hardwire” chromosome-wide gene expression levels of their normal tissue of origin in addition to targeting tissue-specific driver genes.

## Methods

### Tissue and tumor type inclusion

Chromosome arm-wide gain and loss data of each tumor type from the TCGA were obtained from a recent pan-cancer study conducted by Taylor et al. [11], pre-processed cancer gene expression data of each tumor type from the TCGA was obtained from the xena browser (https://tcga.xenahubs.net/) [21], and likewise of the normal tissue of origin of each tumor type was obtained from the GTEx (Genotype Tissue Expression) project portal online (see GTEx Analysis Release V6p at https://www.gtexportal.org/home/datasets) [22]. Clinical stage data of tumor samples was made publicly available from the TCGA Clinical Data Resource (TCGA-CDR) publicly available on the GDC website (https://gdc.cancer.gov/about-data/publications/pancanatlas) [23]. Throughout this study, we worked with pre-processed gene expression data that was quantified in Reads Per Kilobase of transcript, per Million mapped reads (RPKM) by the authors of the respective consortiums with no additional normalization. The RPKM values are unlikely to be confounded by whole genome doubling events as they are already library size normalized, through dividing by the total number of reads in a sample. Furthermore, the GTEx samples were collected from normal individuals, which lack any whole genome duplication events. For analysis comparing tumor types to their normal tissue of origin, data from 25 tumor types with publicly available gene expression data of their normal tissue of origin from GTEx were considered. Likewise, for comparing normal tissue-specific methylation and expression levels, only 11 tumor types which had corresponding publicly available methylation data of their normal tissue of origin were considered. Additional file 1: Table S1, systematically documents for each of the 33 tumor types in the TCGA whether there was an independent publicly available gene expression and methylation dataset of the corresponding normal tissue of origin. Details of publicly available normal tissue methylation datasets that we curated are described below.

### Curation and pre-processing normal tissue-specific methylation datasets

Processed methylation datasets of normal tissues were collected from the Gene Expression Omnibus (GEO) database [24]. For consistency, we restricted our search to datasets where methylation was quantified using the same platform (Illumina 450K). This resulted in the identification of 18 tissue-specific methylation datasets, which were analyzed together (see Additional file 2: Table S2) [25–40]. These were datasets spanning different studies comparing methylation levels of organ tissues between diseased and normal control individuals. We only selected methylation profiles of normal control individuals for further analysis. Moreover, multiple datasets containing samples coming from the same organ tissue were merged to generate one methylation dataset per organ. The methylation data of each dataset was pre-processed in the following steps:

1. Filtering out probes within 15 base pairs of single nucleotide polymorphisms [41].
2. Re-normalizing the beta values between type 1 and type 2 probes using beta mixture quantile normalization [42]. This minimizes biases that may arise due to sensitivity differences between the two probe designs.

### Computation of chromosomal arm imbalance score in cancerous tissues

We used the TCGA sample-wise chromosomal arm gain and loss calls provided in supplementary data of the Taylor et al. [11] study, where the ploidy of each tumor sample was first determined via the ABSOLUTE algorithm [43]. Then, independent chromosome arm copy number alterations were distinguished from whole genome duplication events by comparing the absolute integer copy number of chromosomal arm regions to the baseline tumor ploidy. Each segment was designated as gained, deleted, or neutral compared to the ploidy of each tumor sample. The scores of each arm are − 1 if lost, + 1 if gained, 0 if non-aneuploid, and “NA” otherwise. For sake of consistency, all “NA” entries were re-set to 0 (i.e., we considered those samples non-aneuploid for that arm). Sample-wise chromosome arm gain and loss calls from the METABRIC breast cancer dataset using ABSOLUTE were provided to us upon request from Shukla et al. [44]. The discrete representation was used because it is most fitting to describe arm-level changes, which may be either gained (1) or lost (− 1) by definition, rather than continuous GISTIC data, which is better suited for studying targeted focal copy number alterations.

For each of the 39 chromosomal arms, we defined an arm imbalance score for a set of cancer types sharing the same tissue of origin (or a singular cancer type), by computing the difference between the frequency of gains and losses. Formally:

$$ \mathrm{Arm}\ \mathrm{Imbalance}\ \mathrm{Score}\left({A}_i,{T}_j\right)=\frac{\sum \limits_{\mathrm{samples}\ s\  in\ {T}_j\ }{I}_{sG}\left({A}_i\right)-\sum \limits_{\mathrm{samples}\ s\  in\ {T}_j\ }{I}_{sL}\left({A}_i\right)}{\mathrm{Number}\ \mathrm{of}\ \mathrm{samples}\  in\ {T}_j} $$

where *A*_*i*_ is chromosomal arm *i* (of 1 to 39 chromosomal arms), *T*_*j*_ is the tissue of origin of all tumor types arising from tissue *j*, and the indicators *I*_*sG*_(*A*_*i*_) and *I*_*sL*_(*A*_*i*_) are defined by:

$$ {I}_{sG}\left({A}_i\right)=\left\{\begin{array}{c}1, if\ \mathrm{s}\mathrm{a}\mathrm{mples}\ \mathrm{s}\ \mathrm{has}\ \mathrm{a}\ \mathrm{gain}\ \mathrm{of}\ \mathrm{a}\mathrm{rm}\ {A}_i\\ {}0\ \mathrm{otherwise}\end{array}\right. $$

$$ {I}_{sL}\left({A}_i\right)=\left\{\begin{array}{c}1,\mathrm{if}\ \mathrm{samples}\ s\ \mathrm{has}\ \mathrm{a}\ \mathrm{loss}\  of\ \mathrm{a}\mathrm{rm}\ {A}_i\\ {}0\ \mathrm{otherwise}\end{array}\right. $$

Hence, arms that are more frequently gained are assigned positive scores, while arms that are more frequently lost are assigned negative scores. Arms that are neither gained nor lost and arms where the frequency of gains and losses is comparable are assigned neutral (~zero) score. However, the latter is negligible since chromosome arms that are frequently gained are rarely lost in a specific tumor type and vice versa. This score is hence equivalent to the mean value of gains/loss incidences in set of tumor types considered and chromosomal arm.

### Using permutation tests to evaluate correlations significance

In this study, we computed correlations across cancer/tissue types and across chromosomal arms. To evaluate whether the magnitude of correlations is significant compared to random, we employed a permutation test, to estimate a background null distribution of the number of positive correlations. We therefore repeated 1000 iterations of randomly shuffling the cancer/tissue pairing and 1000 iterations of randomly shuffling the arm-level pairing. We compared the number of positive correlations *P*, achieved with the true pairings to this background (*N*_*i*_, *i* = 1, 2, …, 1000), to compute a *p* value and accept or reject the null hypothesis, denoted as $\frac{\sum \limits_1^{1000}{N}_i>P}{1000}$ .

In a similar manner, we tested whether mean arm-wide gene expression levels of each of the 39 chromosome arms in a sample are informative for predicting the sample’s tissue of origin, compared to the background of any random aggregation of gene expression into 39 groups. Therefore, we designed a permutation test with 1000 iterations. In each iteration, we quantified how accurately we can predict tissue of origin based on randomly aggregating genes into 39 groups with similar sizes as that of chromosomal arm assignment. We evaluated the number of times (out of 1000) in which the multiclass prediction accuracies of the shuffled predictor (*N*_*i*_,with randon aggregation of genes into 39 groups) exceeded the original predictor (*P*, with the aggregation of genes to 39 groups by chromosomal arm), to derive an empirical permutation *p* value, denoted as $\frac{\sum \limits_1^{1000}{N}_i>P}{1000}$.

### Quantile normalization of gene expression and methylation values for cross tissue comparison and visualization

To enable side-by-side comparison and visualization of the arm imbalance scores with mean chromosomal arm mean gene expression levels in different normal tissues (and likewise in different cancers), the gene expression and arm-imbalance values need to be on the same scale. Hence, we additionally quantile-normalized the mean gene expression levels using the chromosomal arm imbalance distribution as reference, to enable visualization by generating similar expression distribution across different tissues. We applied the same approach to quantile normalized chromosome arm-wide mean methylation levels in normal tissues to visualize normal methylation against normal gene expression in each tissue.

### Obtaining chromosome-wide distribution of relevant oncogenes and tumor suppressors in each cancer type

We obtained a comprehensive list of known (or potential) oncogenes and tumor-suppressors driving each cancer type from supplementary data of a recent pan-cancer study conducted by Bailey et al. [13]. This list was obtained from supervised machine learning predictions based on features derived from mutation, copy number, gene expression, and methylation changes observed in genes across different cancer types. Given a cancer type, the oncogenes-tumor suppressor imbalance score for each arm in a given cancer type (or collection of cancer types) was formally defined as follows:

Oncogene-tumor suppressor imbalance score = fraction of driver genes on the arm that are oncogenes − the fraction of driver genes on the arm that are tumor-suppressors.

### Normal and cancer tissue of origin classification and clustering

We classified normal (and likewise, cancer) samples using the chromosomal-arm level expression of those samples. For each sample, we calculated the mean gene expression level of the genes in each chromosomal arm. This resulted in 39 unique features per sample (one per arm). We then performed K-Nearest-Neighbors (KNN based on Euclidean distances, with *K* = 5, the value for which the best performance was observed for cancer type classification from *K* = 3, 5, 7) classification with a Leave-One-Out cross validation (LOOCV), aiming to classify each sample based on the 39 arm level features and calculate the resulting accuracy (percentage of correctly classified samples in the LOOCV). An analogous approach was taken for classification of tissue of origin based on methylation data. Additionally, to rule out potential confounding batch effects in gene expression data and the leave one out cross-validation procedure used, we re-estimated overall KNN performance using 5-fold cross validation (Additional file 13: Table S8). Visualization of the clusters of normal and cancer samples was performed using Rtsne package and default hyper-parameter settings [45]. For performing hierarchical clustering of different tissue types, each tissue type is summarized as a vector of 39 features; one for each arm. Four different hierarchical clustering analyses were performed using “hclust” utility function available in R. For each hierarchical clustering, a different set of 39 features was used. They are systematically listed:

1. Chromosomal arm imbalance score computed across all cancer types originating from the same tissue
2. Mean arm-wide normal gene expression across all genes and all normal samples belonging to the same tissue.
3. Mean arm-wide cancer gene expression across all genes and all samples originating from the same tissue
4. Arm level oncogene-tumor suppressor imbalance score across all cancer types originating from the same tissue

Cophenetic distances between any two hierarchical clusterings were calculated using “cophenetic” utility function available in R.

## Results

### Correlation between frequencies of cancer type-specific aneuploidies and mean chromosome arm-wide gene expression levels of their normal tissue of origin

**Fig. 1:**
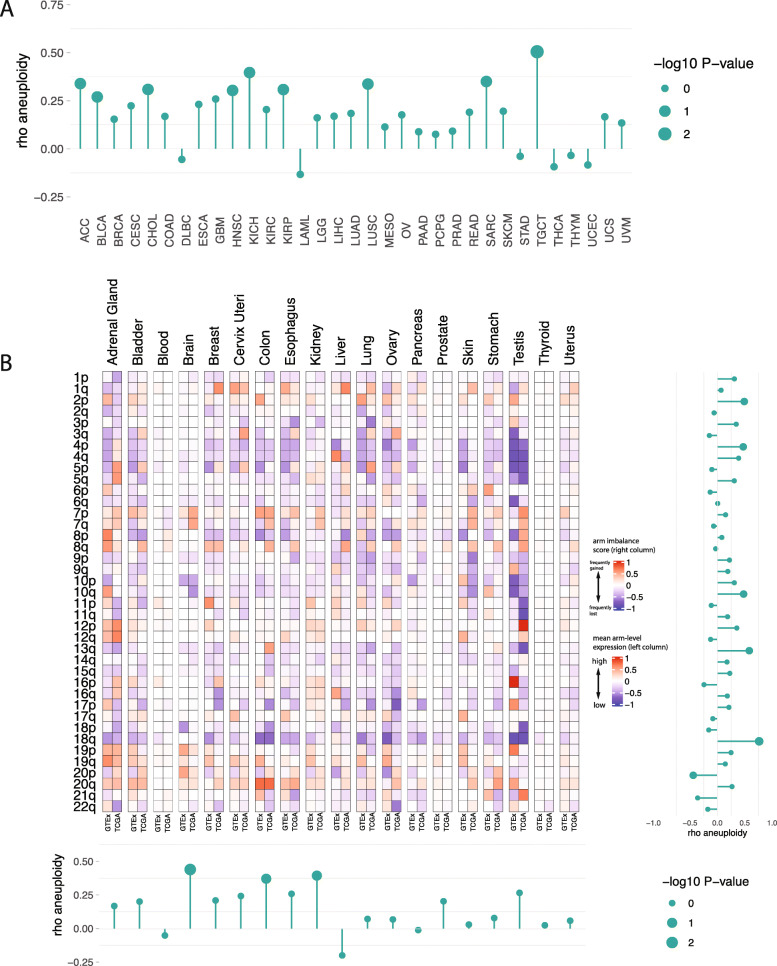
Correlations of chromosome arm-wide gene expression levels and chromosome arm-wide aneuploidies. **a** Correlation plot of chromosome arm-wide gene expression levels in cancers and patterns of chromosome arm-wide gains and losses in cancers reported in the TCGA database. Bar plot represent the Spearman rank correlations for each cancer type independently. The height of the bar reflects the correlation coefficient, and the size of the circle the significance. Size of 2 indicates *p* value < 0.01, size of 1 indicates *p* value < 0.1, and size of 0 indicates *p* value < 1. **b** Correlation of chromosome arm-wide gene expression levels based on the GTEx database (left column) with chromosome arm-wide aneuploidies in associated cancer types based on data reported in the TCGA database (right column), respectively, for 19 tissue entities. The arm imbalance score is reflected in colors: red indicates more frequent gains compared to losses; blue indicates more frequent losses compared to gains. The hue of the colors indicates the frequency of copy number changes and the quantile normalized levels of mean chromosome arm-wide gene expression, respectively. Barplots shown beside each heatmap are the Spearman rank correlations (horizontal bars indicate comparisons for each arm independently; vertical bars indicate comparisons for each tissue independently). A size of 2 indicates *p* value < 0.01, a size of 1 indicates *p* value < 0.1, and size of 0 indicates *p* values < 1. TCGA study abbreviations: ACC, adrenocortical carcinoma; BLCA, bladder urothelial carcinoma; BRCA, breast invasive carcinoma; CESC, cervical squamous cell carcinoma and endocervical carcinoma; CHOL, cholangiocarcinoma; COAD, colon adenocarcinoma; DLBC, diffuse large B cell lymphoma; ESCA, esophageal carcinoma; GBM, glioblastoma multiforme; HNSC, head and neck squamous cell carcinoma; KICH, kidney chromophobe; KIRC, kidney renal clear cell carcinoma; KIRP, kidney renal papillary carcinoma; LAML, acute myeloid leukemia; LGG, low-grade glioma; LIHC, liver hepatocellular carcinoma; LUAD, lung adenocarcinoma; LUSC, lung squamous cell carcinoma; MESO, mesothelioma; OV, ovarian serous cystadenocarcinoma; PAAD, pancreatic adenocarcinoma; PCPG, pheochromocytoma and paraganglioma; PRAD, prostate adenocarcinoma; READ, rectum adenocarcinoma; SARC, sarcoma; SKCM, skin cutaneous melanoma; STAD, stomach adenocarcinoma; TGCT, testicular germ cell tumors; THCA, thyroid carcinoma; THYM, thymoma; UCEC, uterine endometrial carcinoma; UCS, uterine carcinosarcoma; UVM, uveal melanoma

Taylor and colleagues [11] comprehensively recorded for each tumor sample in the TCGA if a specific chromosome arm was gained or lost (while accounting for the baseline tumor ploidy). We used this data to compute the mean chromosome arm imbalance score of each arm in a given cancer type (or collection of cancer types) emerging from the same tissue of origin. In short, this score measures the difference between the frequency of gains and losses of a specific chromosome arm (see the “Methods” section). As a first step, we validated previous observations by showing that the mean gene expression levels over all genes and all samples from the same chromosome arms and cancer type included in the TCGA database, respectively, positively correlate with the corresponding arm imbalance scores (Fig. 1a, Additional file 3: Table S3, Additional file 4: Table S4). This analysis confirmed that genomic copy number alterations in cancer genomes directly affect gene expression levels. We additionally computed chromosome arm imbalance scores in an independent cohort of 1980 breast cancer patients (METABRIC) [44] with publicly available copy number and gene expression data and found consistent trends (Additional file 5: Figure S1A and S1B). After having validated this correlation, we next computed the mean expression levels over all genes and all samples from the same chromosome arm and normal tissue, respectively, from the GTEx database. These values were then correlated with the mean chromosome arm imbalance scores of respective cancer types emerging from that tissue. Figure 1b plots a heatmap with rows indicating chromosome arms. The chromosome arm-wide mean expression levels in each normal tissue and corresponding arm imbalance scores in associated cancer types are juxtaposed and quantile normalized to the same scale for visualization and comparison (Additional file 6: Table S5, Additional file 7: Table S6, Additional file 8: Table S7).

In general, chromosome arms that are most frequently altered are either predominantly gained or lost, across all samples of a cancer type. That is, the gain and loss frequencies of a chromosome do not cancel each other out, resulting in either positive or negative arm imbalance scores across most cancer types. However, there are some notable exceptions (see for example chromosome 13q which has a positive arm imbalance score only in gastrointestinal tumors). Nevertheless, the frequencies of gains and losses vary by tissue of origin and result in varying arm imbalance scores across cancer types. Among the frequently altered chromosome arms, we see that chromosome arms 13q, 18q, 10q, and 2p have the strongest correlations between their normal tissue-specific mean expression levels and arm imbalance scores, and these correlations are positive. When looking at each tissue individually (columns of Fig. 1b), we see the strongest correlations between the normal chromosome-wide mean expression levels and arm imbalance scores for brain, colon, and kidney tissues, and these correlations are also positive. Although the statistical power to assess the significance of these individual correlations is limited, we see that a majority of correlations (both at tissue and arm level) are positive. We evaluated the overall probability of getting so many positive correlations (both at the arm and tissue level), using a permutation test. To this end, we repeated 1000 times of randomly shuffling the chromosomal arm assignments (rows of Fig. 1b) and another 1000 for the tissue assignments (columns of Fig. 1b). We found that similar or higher correlations were found for the shuffled data in less than 5% of the cases, yielding a permutation *p* < 0.05 for both arm-wise and tissue-wise correlations. A more detailed overview of the correlation signals for each tissue (across all arms) and each arm (across all tissues) is provided in Additional file 9: Figure S2 and Additional file 10: Figure S3, respectively. Furthermore, we separately plotted the correlations between normal arm level expression, cancer arm level expression and the arm imbalance scores for 5 cancer normal tissue pairs (Additional file 11: Figure S4, Additional file 5: Figure S1 panels C and D). We additionally repeated this analysis for early stage tumors from the TCGA database (defined as tumors with AJCC stage classification of 0 or 1). Although the number of tumors available for analysis was further reduced, a similar trend of weak, but predominantly positive correlations was observed (Additional file 12: Figure S5).

**Fig. 2:**
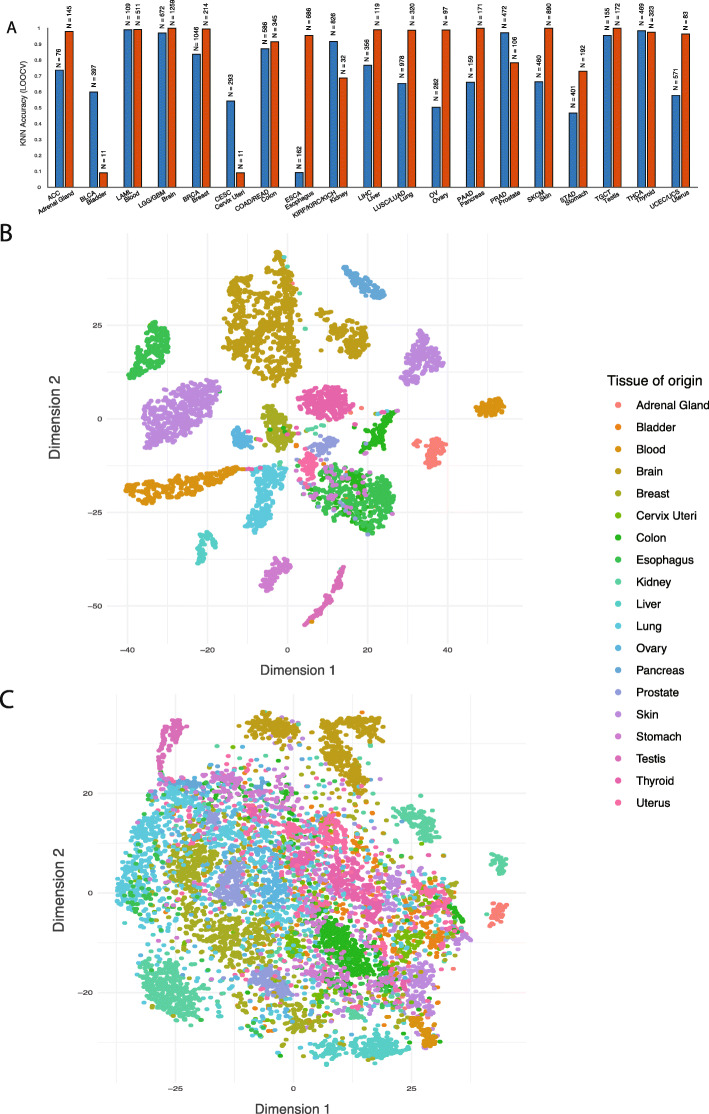
Normal tissue and cancer type classification based on chromosome arm-wide gene expression levels. **a** K-Nearest-Neighbors (KNN) multi-class analysis: predictions made in a leave one out fashion (i.e., the accuracy). Height of bars indicates the fraction of correctly predicted cases. The numbers on top of each bar indicate the number of samples available for each class. **b**, **c** t-Distributed Stochastic Neighbor Embedding (t-SNE) dimensionality reduction analysis of chromosome arm-wide mean gene expression levels in normal tissues (**b**) and in cancers (**c**)

If certain chromosome arm aneuploidies might “hard-wire” the chromosome arm-wide gene expression levels specific to their normal tissues, one should be able to classify the tissue of origin of normal and cancer tissue samples just based on the mean chromosome arm-wide gene expression levels of each of the 39 arms. To test this hypothesis, we obtained the mean gene expression levels for each arm in each normal tissue sample in GTEx (and likewise for each cancer sample in TCGA) resulting in 39 unique features. Then K-Nearest Neighbors (K-NN) multi-class classification was applied with leave-one-out cross validation (see the “Methods” section for more details). We find that mean chromosome arm-wide gene expression can effectively classify the tissue of origin of both normal and cancer samples from GTEx and TCGA, respectively, and that the performance is generally better for normal tissues (Fig. 2a, Additional file 13: Table S8, Additional file 14: Table S9). The resulting accuracy was better for tissues with higher case numbers, as expected for KNN analyses. Furthermore, these results could never be obtained when the chromosome assignment of genes was randomly shuffled (by repeating 1000 shuffling of the chromosomal assignments of genes, permutation test *p* value < 0.001). To rule out the possibility that the results are inflated because of the leave-one-out cross validation technique, we performed a fivefold cross validation analysis confirming our results (Additional file 15: Table S10). To visualize these classifications, we used t-distributed Stochastic Neighbor Embedding (t-SNE) dimensionality reduction of the 39-dimensional feature space. We found that samples from the same normal tissues cluster closely in most cases (Fig. 2b), but to a lesser extent for cancer entities (Fig. 2c). The separate sub-clusters within each tissue correspond to the different anatomical regions of the tissues that were sampled from GTEx. Overall, these results suggest that certain chromosomal aneuploidies acquired by tumors might hardwire tissue-specific gene expression levels of their tissue of origin.

### Correlation between frequencies of cancer type-specific aneuploidies and the tissue-specific oncogenes and tumor suppressors that reside on the respective chromosomes

**Fig. 3:**
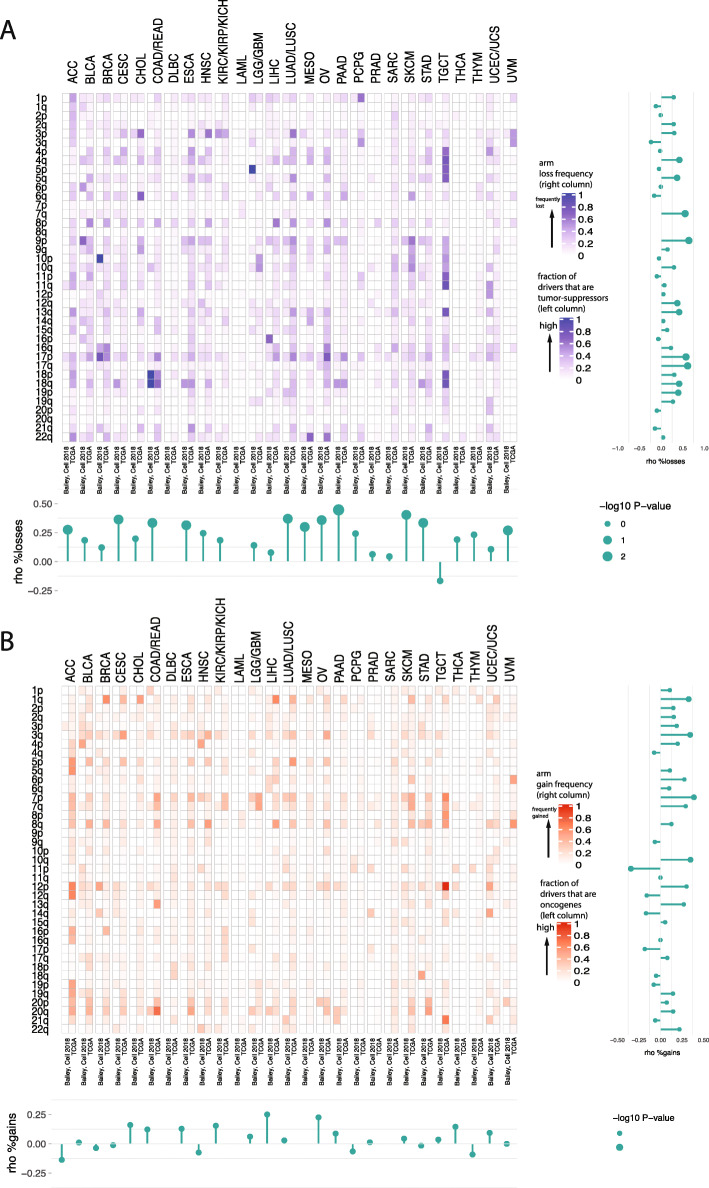
Distribution of cancer driver genes and chromosome arm-wide aneuploidies. For each set of cancer types with shared tissue of origin, we plot: **a** the fraction of driver genes on each arm that are considered to be tumor suppressors (left column) and the frequency of losses reported for the arm. The bluer the color, the higher the tumor suppressor burden (and likewise for the frequency of losses. **b** The fraction of driver genes on each arm that are considered to be oncogenes (left column) and the frequency of gains reported for the arm (right column). The redder the color, the higher the oncogenic burden (and likewise for frequency of gains). Barplots shown beside each heatmap are the Spearman rank correlations (horizontal bars indicate comparisons for each arm independently; vertical bars indicate comparisons for each tissue independently). The size of bubbles indicates the *p* value. A size of 2 indicates *p* value < 0.01, a size of 1 indicates *p* value < 0.1, and size of 0 indicates *p* values < 1. As seen at the tissue level, correlation between tumor suppressor burden and frequency of losses is almost always positive (empirical *p* value after randomly shuffling data < 0.05), whereas that is not the case for gains

**Fig. 4:**
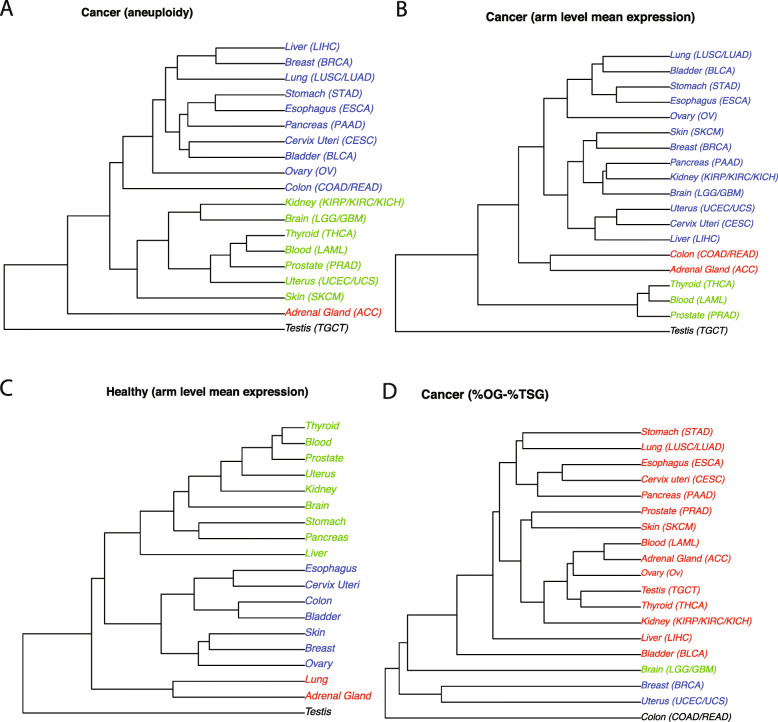
Hierarchical cluster analysis of cancers and normal tissues. **a** Cancer chromosome arm-wide gains and losses, **b** cancer mean chromosome arm-wide gene expression, **c** mean chromosome arm-wide gene expression of normal tissues, and **d** chromosome arm-wide imbalance of tumor suppressor genes and oncogenes. Note that the clusters are similar in **a**–**c**, yet different in **d**

Recent studies have looked at the connection between specific chromosomal gains and losses and driver genes located on these chromosomes for specific cancer types [12, 46]. Here, we revisited this connection. For each tissue analyzed in this study, the correlation between the frequency of losses in associated cancer types and the fraction of drivers that are tumor suppressors is consistently strong and positive (Fig. 3a, permutation test with 1000 random shufflings of arms and tissue pairing of the values in Fig. 3a, *p* value < 0.05; see the “Methods” section, Additional file 16: Table S11, Additional file 17: Table S12, Additional file 18: Table S13, Additional file 19: Table S14). The strongest of these correlations were observed for chromosome arms 17p, 17q, and 9p. The direction of correlation between gains of chromosome arms and the location of tissue-specific oncogenes is, however, less clear (Fig. 3b, permutation test *p* value after random shuffling> 0.05, Additional file 6: Table S5). To explore this further, we performed four hierarchical clustering analyses of tissues based on (i) chromosomal arm imbalance scores in associated cancer types (Fig. 4a), (ii) mean chromosome arm-wide gene expression levels in associated cancer types (Fig. 4b), (iii) mean chromosome arm-wide gene expression levels in normal tissues (Fig. 4c), and (iv) chromosome arm-wide imbalance in the fraction of oncogenes and tumor suppressor genes originating from each tissue (Fig. 4d). For ease of visualization, the tissues were partitioned and colored by 4 distinct clusters obtained from each hierarchical clustering separately. To further systematically quantify the similarities between two clusterings, we computed the Spearman correlation between cophenetic distances defined by each clustering. We found that the cophenetic distances among tissues based on chromosomal arm imbalance scores (Fig. 4a) and mean chromosome arm-wide normal gene expression levels (Fig. 4c) are highly similar (Spearman correlation r = 0.61, *p* value = 2.2E−16). Likewise, a strong Spearman correlation (*r* = 0.52, *p* value = 2.997E−13) was obtained when comparing cophenetic distances based on arm imbalance scores (Fig. 4a) and mean chromosome arm-wide cancer gene expression levels (Fig. 4b). However, the Spearman correlation between cophenetic distances of tissues based on arm imbalance scores (Fig. 4a) and distribution of tissue-specific oncogenes and tumor suppressor genes (Fig. 4d) is − 0.09, with a *p* value = 0.2067. While the list of tissue-specific cancer driver genes is still incomplete, these results suggest that copy number changes in resident driver genes may not be sufficient to explain the observed tissue-specificity of chromosomal aneuploidies in cancers.

### Samples from the same normal tissue also cluster together by their mean chromosome arm-wide methylation levels

**Fig. 5:**
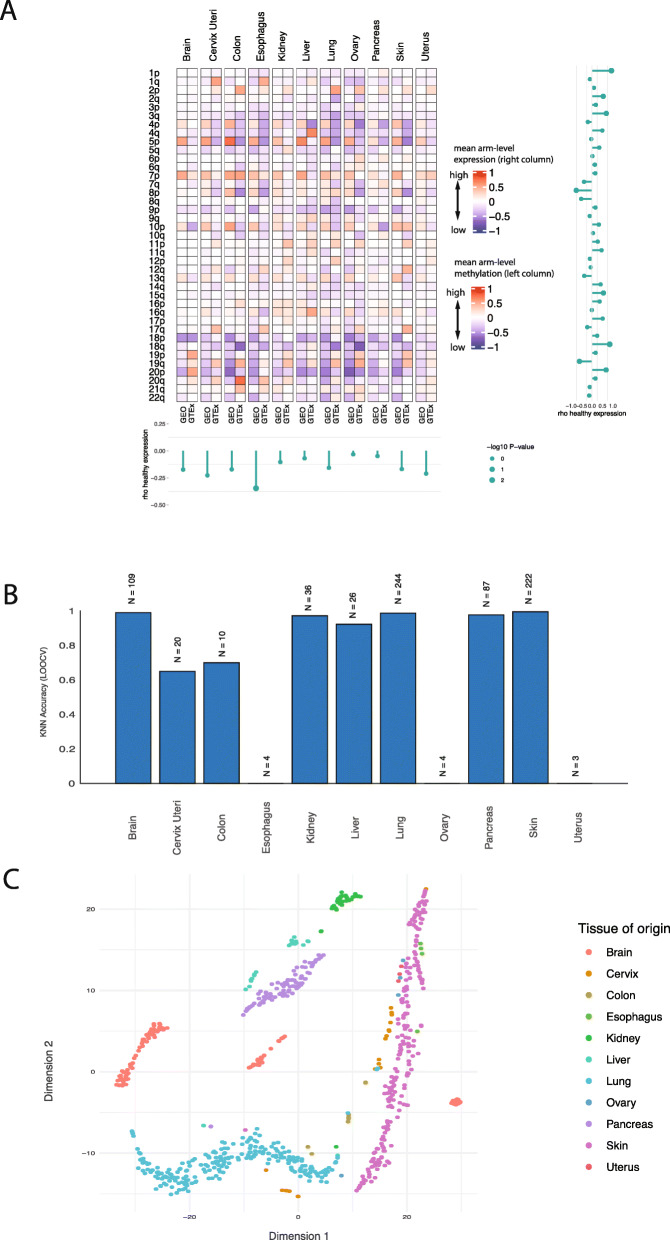
Correlation of chromosome arm-wide methylation levels and chromosome arm-wide gene expression. **a** For each tissue with available normal methylation data, we plot the mean arm-wide methylation levels of each arm (left column) and the mean arm-wide expression levels of each arm (right column). The mean expression and methylation values are quantile normalized to the same scale (see the “Methods” section) for comparison and visualization. For left column: the redder the color, the higher the arm-wide methylation level; the bluer the color, the lower the arm-wide methylation level. For right column: the redder the color, the higher the arm-wide expression level; the bluer the color, the lower the arm-wide expression levels. Bar plots besides the heatmap are Spearman rank correlations (horizontal bars indicate comparison for each arm independently; vertical bars indicate comparison for each tissue independently). The size of bubbles indicates the *p* value. A size of 2 indicates *p* value < 0.01, a size of 1 indicates *p* value < 0.1, and size of 0 indicates *p* values < 1. As seen at the tissue level, correlation between arm-wide methylation levels and expression levels is consistently negative (empirical *p* value after random shuffling the data < 0.05). **b** Leave One Out Cross-Validation Accuracy of predicting each tissue entity based on chromosome wide mean methylation levels of each sample. The height of the bar indicates the accuracy quantified as fraction of samples correctly classified. The numbers on top of each bar indicate the number of samples from a given tissue. **c** tSNE plot depicting the clustering of different tissue samples by chromosome arm-wide mean methylation levels

A possible mechanism regulating chromosome-wide gene expression levels in normal tissues is DNA methylation. Therefore, in a fashion similar to Fig. 1b, we explored whether mean chromosome arm-wide methylation levels correlate with the mean chromosome arm-wide gene expression levels. The Gene Expression Omnibus (GEO) database provides genome-wide methylation levels for 11 different tissue types, all obtained using the same Illumina 450K platform (Additional file 2: Table S2). Based on these data, we analyzed chromosome arm-wide mean methylation patterns for 11 tissues from 765 samples (Methods, Additional file 20: Table S15). For each tissue, we observed that differences in mean methylation levels across chromosome arms within a tissue are consistently negatively correlated with corresponding mean arm-wide gene expression levels (permutation test with 1000 random shufflings of arms and tissue pairing of the values, *p* value < 0.05, see the “Methods” section) (Fig. 5a, Additional file 7: Table S6). However, for a single arm across tissues, the directionality of correlations is less consistent. This could potentially be due to the small number of tissues analyzed. Furthermore, an individual sample-level classification analysis using the KNN algorithm revealed that one can predict (in leave one out cross-validation) the normal tissue of origin of individual samples just based on chromosome arm-wide mean methylation levels. The clustering of samples by tissue is visualized using t-SNE dimensionality reduction. (Fig. 5b, c, Additional file 21: Table S16). Tissues with very few samples had poor classification accuracy as expected from KNN. These results suggest that normal chromosome arm-wide methylation levels may play some part in regulating the transcriptional output of each chromosome arm.

## Discussion

Chromosomal aneuploidies are a defining feature of tumors of epithelial origin. These aneuploidies result in tumor type-specific genomic imbalances [1, 4–6, 10]. As of yet, there is no sufficient explanation for this specificity [6]. In this work, we systematically compared the frequencies of chromosome arm gains and losses in different cancer types to the mean chromosome arm-wide gene expression levels in normal tissues of origin and distribution of known or implicated tissue-specific oncogenes/tumor suppressors across chromosome arms. Our analysis revealed a complex picture of factors driving frequent chromosome arm copy number changes in specific cancer types. Specifically, we notice recurrent losses in chromosome arms in cancer types where tissue-specific tumor suppressors reside, suggesting that these losses broadly target these driver genes. However, the targets of recurrent tissue-specific chromosomal gains are less clear. While it is possible that these chromosomal gains are targeting yet unidentified oncogenes, our analysis of normal chromosome-wide gene expression and methylation data suggests an alternative paradigm in which these alterations instead aim to hardwire gene expression levels of normal tissue origin. This notion is further supported by recent observations across multiple cancer types where oncogenes were found to be preferentially activated via extra-chromosomal DNA [47].

The functional implications of many genes that are affected by these alterations remain incompletely understood. We previously showed experimentally that the gain of chromosome 13 in colorectal cancer activates both Notch and Wnt signaling [48] and that the acquisition of extra copies of chromosome 7 in normal colon cells results in upregulation of cancer-associated pathways [49], which could imply that tissue type-specific chromosome arm-wide gene expression levels promote cellular fitness. Of note, Sack et al. [50] have demonstrated that the inclusion of tissue-specific growth promoting genes strengthens the correlation between chromosome arm loss/gain ratios and the proliferation-driving capability of each chromosome arm in breast and pancreatic cancers. Graham and colleagues reported a general role of copy number alterations and metabolic selection pressure [51]. Despite the ubiquitous presence of chromosomal aneuploidies in most solid tumors, there are also several publications pointing to a reduction of cellular fitness as a consequence of general aneuploidy in model systems such as yeast, immortalized murine embryonic fibroblasts, and typically near-diploid cancer cells engineered to harbor specific trisomies [52–54], so the functional implications of these events remains an open challenging question.

There are some limitations specific to the data analysis conducted in this study. Firstly, our analyses comparing cancer types to normal tissues were restricted to tissues where data was measured in a homogeneous fashion on the same platform and publicly available (i.e., GTEx for gene expression and GEO for methylation). Furthermore, we restricted ourselves to external data sources for normal tissue expression and methylation rather than use adjacent normal tissue samples from the TCGA. This was mainly due to incomplete availability of methylation and expression of normal adjacent to tumor samples for many cancer types and the presence of stromal and immune cell contamination in these tissues [55, 56]. Secondly, identification of existing and potentially new cancer type-specific oncogenes and tumor suppressors was previously done by combining evidence from multi-omic sources into one prediction score using supervised machine learning [13]. However, this list is still incomplete and the mechanism of action of many of these genes in different cancer types is not completely understood. Thirdly, since we were exploring correlation patterns across different tissues and cancer types, it is likely that more significant associations would be observed in arms with specific, high-intensity trends of either gain or loss compared to arms that are less frequently altered.

## Conclusions

**Fig. 6:**
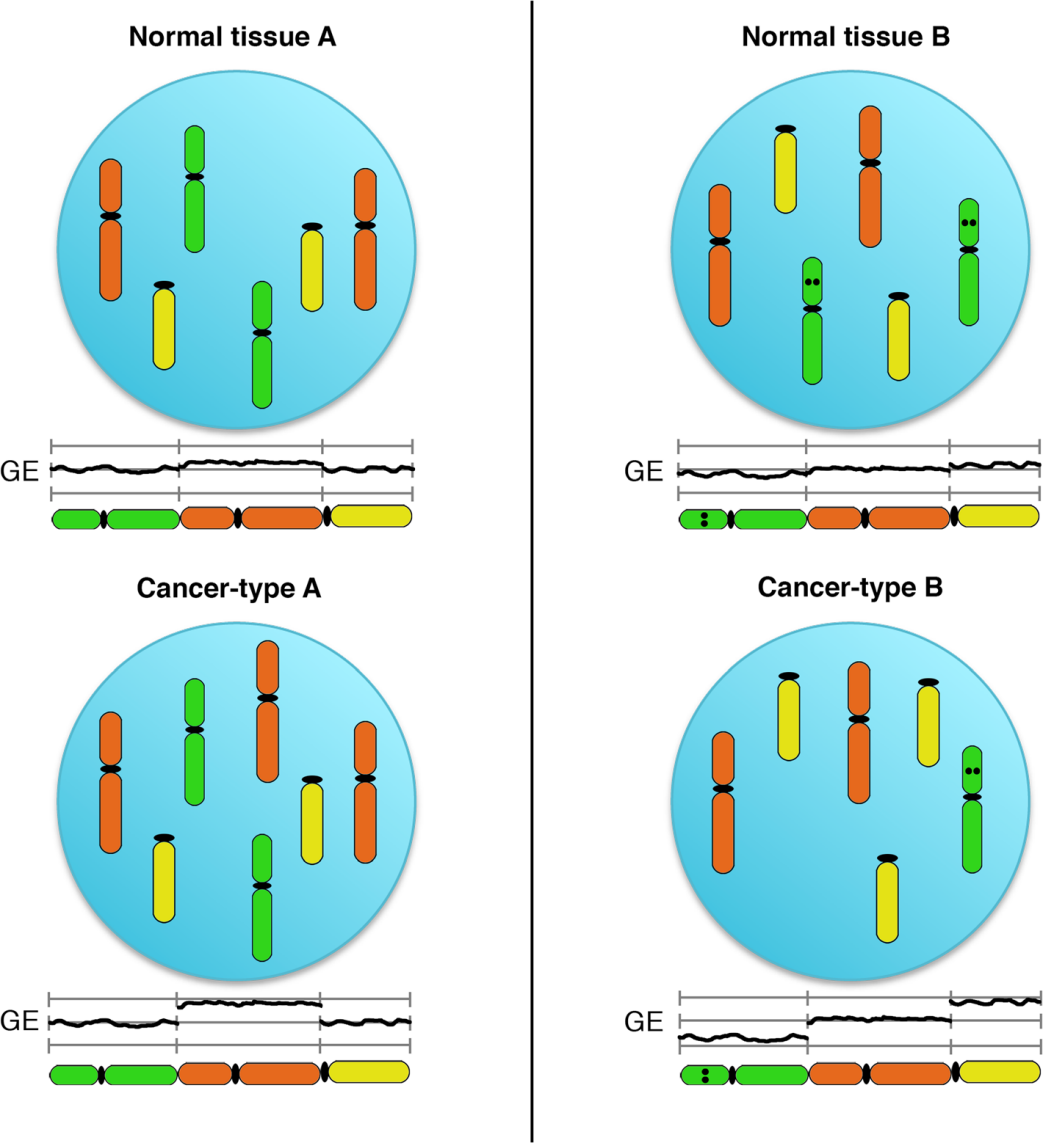
Schematic presentation of the results. Genes on the red chromosomes are expressed at slightly higher levels compared to other chromosomes in normal tissue A, whereas in normal tissue B, the yellow chromosomes shows increased tissue-specific expression and genes on the green chromosome are expressed at lower levels. This results in a subtle increase or decrease in chromosome arm-wide gene expression levels, respectively. The acquisition of chromosomal aneuploidies in the respective cancer types (gain of the red chromosome in cancer type A and the yellow chromosome in cancer type B, accompanied by the loss of the green chromosome in cancer type B) amplifies this effect and provides the genetic basis of “hard-wiring” tissue-specific chromosome arm-wide gene expression levels as the basis for clonal expansion. The dots on the green chromosome reflects the presence of a tumor suppressor gene

In summary, our data analysis suggests that chromosome aneuploidies could be potentially involved in the maintenance of gene expression levels characteristic of the normal tissue of origin of cancers, in addition to targeting cancer type-specific driver genes (Fig. 6).

## Supplementary Information

**Additional file 1: Table S1.** Mapping of cancer type barcodes from TCGA to normal tissue of tumor origin in external data sources.

**Additional file 2: Table S2.** Manually curated DNA methylation datasets of different non cancerous tissues.

**Additional file 3: Table S3.** Mean gene expression levels of each chromosome arm in each cancer type (from TCGA).

**Additional file 4: Table S4.** Chromosome arm imbalance score (i.e., frequency of gains - frequency of losses) of each chromosome arm in each cancer type.

**Additional file 5: Figure S1.** Scatter plots correlating chromosome arm imbalance scores (X axes) with arm-wide expression levels in cancer (upper row, Y axes) and normal tissue (lower row, Y-axes).

**Additional file 6: Table S5.** Mean gene expression levels of each chromosome arm in each normal tissue (from GTEx).

**Additional file 7: Table S6.** Chromosome arm imbalance score (i.e., frequency of gains - frequency of losses) of each chromosome arm in cancer types grouped by normal tissue of tumor origin.

**Additional file 8: Table S7.** Mean gene expression levels of each chromosome arm in cancer types grouped by normal tissue of tumor origin (from TCGA).

**Additional file 9: Figure S2.** Scatter plots showing the relationship between chromosome arm imbalance scores in cancer (Cancer_AN) and normal gene expression (Normal_GE) for each tissue/tumor type.

**Additional file 10: Figure S3.** Scatter plots showing the relationship between chromosome arm imbalance scores in cancer (Cancer_AN) and normal gene expression (Normal_GE) for each chromosome arm.

**Additional file 11: Figure S4.** Scatter plots depicting correlation of chromosome arm-wide gene expression levels in normal tissues based on the GTEx database with chromosome arm wide aneuploidies and corresponding chromosome arm-wide cancer gene expression levels in associated cancer types of 4 tissues (Kidney, Brain, Cervix and Colon).

**Additional file 12: Figure S5.** Correlation of chromosome arm-wide gene expression levels based on the GTEx database (left column) with chromosome arm wide aneuploidies in associated cancer types diagnosed at early stages based on data reported in the TCGA (right column), respectively.

**Additional file 13: Table S8.** Mean gene expression level (in RPKM) of each chromosome arm in each normal tissue sample (from GTEx).

**Additional file 14: Table S9.** Mean gene expression level (in RPKM) of each chromosome arm in each cancer sample (from TCGA).

**Additional file 15: Table S10.** Five fold cross-validation accuracy of KNN classifier based on 39 chromosome arm-wide expression features.

**Additional file 16: Table S11.** Fraction of all cancer driver genes on each chromosome arm that are considered as oncogenes in cancer types from a given tissue of origin.

**Additional file 17: Table S12.** Frequency of gains of each chromosome arm in cancer types grouped by normal tissue of tumor origin.

**Additional file 18: Table S13.** Fraction of all cancer driver genes on each chromosome arm that are considered as tumor suppressors in cancer types from a given tissue of origin.

**Additional file 19: Table S14.** Frequency of losses of each chromosome arm in cancer types grouped by normal tissue of tumor origin.

**Additional file 20: Table S15.** Mean methylation levels of each chromosome arm in each normal tissue.

**Additional file 21: Table S16.** Mean methylation levels (beta values) of each chromosome arm in each normal tissue sample.

## Data Availability

All data generated or analyzed during this study are included in this published article and its supplementary information files. Source data for reproducing Fig. 1 panel A, Supplementary Figure S1 panels A and C is available in Additional file 3: Table S3 and Additional file 4: Table S4. Source data for reproducing Supplementary Figure S1 panels B and D (the METABRIC cohort) were made available to us upon request from Shukla et al. [44]. Source data for reproducing Fig. 1 panel B, Supplementary Figures S2, S3 and S4 are available in Additional file 6: Table S5, Additional file 7: Table S6, Additional file 8: Table S7. Clinical stage data for reproducing Supplementary Figure S5 are publicly available from the TCGA Clinical Data Resource (TCGA-CDR) available at https://gdc.cancer.gov/about-data/publications/pancanatlas [23]. Source data for reproducing Fig. 2 are available in Additional file 13: Table S8 and Additional file 14: Table S9. Source data for reproducing Fig. 3 are available in Additional file 16: Table S11, Additional file 17: Table S12, Additional file 18: Table S13, Additional file 19: Table S14. Source data for reproducing Fig. 4 are available in Additional file 6: Table S5, Additional file 7: Table S6, Additional file 8: Table S7, Additional file 16: Table S11 and Additional file 18: Table S13. Source data for reproducing Fig. 5 are available in Additional file 20: Table S15 and Additional file 21: Table S16.
