# Supplementary material for: Hard wiring of normal tissue-specific chromosome-wide gene expression levels is an additional factor driving cancer type-specific aneuploidies
Source: Genome Med. 2021 May 25;13:93. doi: 10.1186/s13073-021-00905-y (PMC8147418; doi:10.1186/s13073-021-00905-y)
Supplement: Supplementary file 5 — Additional file 5: Figure S1. Scatter plots correlating chromosome arm imbalance scores (X axes) with arm-wide expression levels in cancer (upper row, Y axes) and normal tissue (lower row, Y-axes). [file 13073_2021_905_MOESM5_ESM.docx]

**Additional file 5: Fig. S1:** Scatter plots correlating chromosome arm imbalance scores (X axes) with arm-wide expression levels in cancer (upper row, Y axes) and normal tissue (lower row, Y-axes). The expression values for the different cohorts are on different scales because of platform differences. For ease of visualization, they have been quantile normalized to be on the same scale as that of arm-imbalance scores. Panel’s A and C depict the original reported correlations based on chromosome aneuploidy data from TCGA-BRCA cohort. Panels B and D depict the correlations based on chromosome aneuploidy data from the METABRIC cohort.
