# Supplementary material for: Hard wiring of normal tissue-specific chromosome-wide gene expression levels is an additional factor driving cancer type-specific aneuploidies
Source: Genome Med. 2021 May 25;13:93. doi: 10.1186/s13073-021-00905-y (PMC8147418; doi:10.1186/s13073-021-00905-y)
Supplement: Supplementary file 9 — Additional file 9: Figure S2. Scatter plots showing the relationship between chromosome arm imbalance scores in cancer (Cancer_AN) and normal gene expression (Normal_GE) for each tissue/tumor type. [file 13073_2021_905_MOESM9_ESM.docx]

**Additional file 9: Fig. S2:** Scatter plots showing the relationship between cancer aneuploidy (Cancer_AN) and normal gene expression (Normal_GE) for each tissue/tumor type. Cancer_AN represents the arm imbalance score, Normal_GE reflects the mean chromosome arm wide gene expression. Dots represent the 39 chromosome arms.
