# Supplementary material for: Hard wiring of normal tissue-specific chromosome-wide gene expression levels is an additional factor driving cancer type-specific aneuploidies
Source: Genome Med. 2021 May 25;13:93. doi: 10.1186/s13073-021-00905-y (PMC8147418; doi:10.1186/s13073-021-00905-y)
Supplement: Supplementary file 10 — Additional file 10: Figure S3. Scatter plots showing the relationship between chromosome arm imbalance scores in cancer (Cancer_AN) and normal gene expression (Normal_GE) for each chromosome arm. [file 13073_2021_905_MOESM10_ESM.docx]

**Additional file 10: Fig. S3:** Scatter plots showing the relationship between cancer aneuploidy (Cancer_AN) and normal gene expression (Normal_GE) for each chromosome arm. Cancer_AN represents the arm imbalance score, Normal_GE reflects the mean chromosome arm wide gene expression. Dots represent the 25 tumor types analyzed in our study, which correspond to 19 different normal tissues of origin.
