# Supplementary material for: Hard wiring of normal tissue-specific chromosome-wide gene expression levels is an additional factor driving cancer type-specific aneuploidies
Source: Genome Med. 2021 May 25;13:93. doi: 10.1186/s13073-021-00905-y (PMC8147418; doi:10.1186/s13073-021-00905-y)
Supplement: Supplementary file 11 — Additional file 11: Figure S4. Scatter plots depicting correlation of chromosome arm-wide gene expression levels in normal tissues based on the GTEx database with chromosome arm wide aneuploidies and corresponding chromosome arm-wide cancer gene expression levels in associated cancer types of 4 tissues (Kidney, Brain, Cervix and Colon). [file 13073_2021_905_MOESM11_ESM.docx]

**Additional file 11: Fig. S4:** Panels A,D,G,J depict correlations between arm-level expression in healthy tissue (on Y axis) and the arm imbalance scores in cancer (on X axis) for Kidney, Brain, Cervix and Colon tissues, respectively. Panels B,E,H,K depict correlations between arm-level expression in cancer (on Y axis) and the arm imbalance scores in cancer (on X axis) for Kidney, Brain, Cervix and Colon tissues, respectively. Panels C, F, I, L depict correlations between arm-level expression in healthy tissue (on Y axis) and the arm-level expression in cancer (on X axis) for Kidney, Brain, Cervix and Colon tissues, respectively. While we show scatter plots for 4 tissues, the correlations are predominantly positive for all tissues (permutation test p-value < 0.05). Arm expression levels were quantile normalized to be on the same scale as that of arm imbalance scores for ease of visualization of scatter plots.
