# Supplementary material for: Hard wiring of normal tissue-specific chromosome-wide gene expression levels is an additional factor driving cancer type-specific aneuploidies
Source: Genome Med. 2021 May 25;13:93. doi: 10.1186/s13073-021-00905-y (PMC8147418; doi:10.1186/s13073-021-00905-y)
Supplement: Supplementary file 12 — Additional file 12: Figure S5. Correlation of chromosome arm-wide gene expression levels based on the GTEx database (left column) with chromosome arm wide aneuploidies in associated cancer types diagnosed at early stages based on data reported in the TCGA (right column), respectively. [file 13073_2021_905_MOESM12_ESM.docx]

**Additional file 12: Fig. S5:** Correlation of chromosome arm-wide gene expression levels based on the GTEx database (left column) with chromosome arm wide aneuploidies in associated cancer types diagnosed at early stages based on data reported in the TCGA (right column), respectively, for 11 tissue entities. The arm imbalance score is reflected in colors: Red indicates more frequent gains compared to losses; blue indicates more frequent losses compared to gains. The hue of the colors indicates the frequency of copy number changes and the quantile normalized levels of mean chromosome arm-wide gene expression, respectively. Barplots shown beside each heatmap are the spearman rank correlations (horizontal bars indicate comparisons for each arm independently, vertical bars indicate comparisons for each tissue independently). A size of 2 indicates p-value < 0.01, a size of 1 indicates p-value < 0.1 and size of 0 indicates p-values < 1.
